# INTegRated InterveNtion of pSychogerIatric Care: real-world application and implementation of an advanced integrated telehealth system incorporating machine learning

**DOI:** 10.3389/fpsyg.2025.1696407

**Published:** 2026-01-14

**Authors:** Rigas F. Soldatos, Dimitrios Kasselimis, Christina Parpoula, Eleni Konidari, Vassilis Dimitriou, Everina Katirtzoglou, Dimitris Kiosses, Konstantinos Tsibanis, Anastasia Konsta, Theofanis Vorvolakos, Panagiotis Alexopoulos, Antonios Politis

**Affiliations:** 1 First Department of Psychiatry, National and Kapodistrian University of Athens Medical School, Eginition Hospital, Athens, Greece; 2 Department of Psychology, Panteion University of Social and Political Sciences, Athens, Greece; 3 Neuropsychology and Language Disorders Unit, First Neurology Department, Eginition Hospital, School of Medicine, National and Kapodistrian University of Athens, Athens, Greece; 4 Mental Health Services, School of Health Sciences, University of Patras, Patras, Greece; 5 Weill Cornell Institute of Geriatric Psychiatry, Weill Medical College of Cornell University, White Plains, NY, United States; 6 E-learning Services, National and Kapodistrian University of Athens, Athens, Greece; 7 Department of Psychiatry, School of Health Sciences, University of Thessaloniki, Thessaloníki, Greece; 8 Department of Psychiatry, School of Health Sciences, University General Hospital of Alexandroupolis, Democritus University of Thrace, Alexandroupolis, Greece; 9 Mental Health Services, University General Hospital of Patras, Patras, Greece; 10 Department of Medicine, School of Health Sciences, University of Patras, Patras, Greece

**Keywords:** dementia, low resource areas, machine learning, remote areas, telehealth, Telepsychiatry, telepsychology

## Abstract

**Introduction:**

Older individuals who suffer from mental disorders may encounter accessibility difficulties related to factors such as remoteness and socioeconomic status. The present analysis provides empirical evidence from the INTegRated InterveNtion of pSychogerIatric Care (INTRINSIC) and shows that this network could aid towards the incorporation of tele-psychiatry and tele-neuropsychology into primary healthcare. We propose that such integration, situated within comprehensive health digitalization initiatives, represents a scalable approach to expanding mental health access.

**Methods:**

1,143 individuals from 2022 to 2025, from 11 different sites of INTRINSIC were recruited. Data collection was facilitated via the HEllenic Remote MEntal health Services for old-age (HERMES) Digital Platform, including demographic information, Mini-Cog scores, as well as information based on the Old Age Behavioral Risk Factor Surveillance System (OLA-BRFSS). A machine learning (ML) model was developed, trained, and evaluated using nested cross-validation. The classification analysis outcome was the Mini-Cog scores and eighty-three known risk factors were analyzed. Features were selected using Elastic Net regularization. A Random Forest classifier was then trained on the selected feature, and was employed to classify individuals into two Mini-Cog cognitive performance groups.

**Results:**

The ML algorithm employed in this study revealed eight features to be positively associated with low Mini-Cog scores, including subjective complaints of cognitive problems, retirement, polypharmacy, and history of falls. Five variables demonstrated a positive association with higher Mini-Cog scores, including prior diagnosis of an anxiety disorder, insomnia, and physical exercise. The model achieved a ROC-AUC of 0.76 (Figure 3 and Table 4), with a BAC of 0.65.

**Discussion:**

The present paper presents the first large-scale study on INTRINSIC, including multiple sites and integrating psychiatric, cognitive, medical, as well as sociodemographic variables in state-of-the-art ML models. Our results add to the existing literature on the complex interrelationships of factors affecting cognitive status in older individuals. We propose that INSTRINSIC may function as a benchmark for integrating psychiatric and neuropsychological services within primary healthcare settings, thereby addressing disparities in access to care and diagnostic equity.

## 1 Introduction

Adults affected by age-related brain disorders are expected to increase significantly in the next decades. Mental disorders that affect older people (Skoog, 2011; Mendez and Cummings, 2003) pose detrimental effects on overall quality of life (Banerjee et al., 2006), adding to the increased prevalence of depression among older individuals (Abdoli et al., 2022). The interplay between mood disorders and pathological cognitive decline in dementia and Mild Cognitive Impairment (MCI) has been well-established (Bennett and Thomas, 2014; Rubin, 2018; Petersen and Negash, 2008), with genetic polymorphisms being connected to the observed comorbidity between Alzheimer’s Disease and Major Depressive Disorder (Brzezińska et al., 2020). Emerging evidence has revealed a complex framework of interaction between cognition, mood, and health status (Alexopoulos, 2005; Taylor, 2014). It has been argued that healthy aging can also result in cognitive slowing and difficulties in emotional regulation, which may lead to changes and challenges in everyday life (Woodard et al., 2012; La Corte et al., 2016; Pettigrew and Martin, 2014). There are other neurological and/or psychiatric disorders that can cause subtle cognitive deficits, such as cerebral microbleeds (Rosenbaum Halevi et al., 2019), leukoencephalopathy (Boyle et al., 2016; Van Den Berg et al., 2018), multiple sclerosis (Katsari et al., 2020), as well as anxiety disorders and/or mood disorders (Ma, 2020). Identifying “normal aging” may be one of the greatest challenges in modern clinical practice (Pertl et al., 2017; Poirier et al., 2021). Contemporary literature shows that any cognitive or psychological changes demonstrated by older individuals should be examined in a multifactorial framework, integrating biological, behavioral and psychosocial variables (Harada et al., 2013; Deary et al., 2009).

There are three main barriers of a multifactorial framework: geographical disparities of psychogeriatric services, reduced accessibility and resource allocation. Recent systematic reviews confirmed that the prevalence of cognitive decline in rural areas is higher than the prevalence in urban ones (Xue et al., 2018; Mollalo et al., 2025). The obstacles to healthcare access are widespread in old populations, with the consequence that old people with mental disorders and cognitive decline do not benefit from specialized mental and cognitive health services. The need to increase accessibility and methods of optimization has been suggested, which could eventually lead to equity (Levesque et al., 2013; Wang, 2012). In Greece, older patients with mental disorders such as depression and cognitive decline face great difficulties accessing specialized services and mental and cognitive health services are lacking in remote and rural areas. In terms of resources, there is an imbalance worldwide in mental health care resources between urban and rural areas. Greece has been examined in the past in a large-scale comparative study on European healthcare systems, and was found to have the lowest public health expenditure (as a percentage of the total health expenditure) and the highest out-of-pocket payments, among European countries (Wendt, 2009). It should be also noted that neuropsychology is a quite new discipline in Greece (Liozidou et al., 2023) and has not yet been incorporated into the national health system.

Taking into account that remoteness and insularity undermine access to older adults mental and cognitive health services, tele-health practices should be considered as a facilitator of remote assessment and treatment. Several attempts towards this goal have been made. Research shows that telepsychogeriatrics is of comparable efficacy compared to face-to-face treatment (Hagi et al., 2023) and is of high acceptability among clinicians and beneficiaries (Abuyadek et al., 2024; Sharma and Devan, 2023). Several researchers argue in favor of remote cognitive assessment via tele-neuropsychology, given the accessibility difficulties discussed above (Hewitt et al., 2022; Sperling et al., 2024). Data seem promising, showing efficacy of teleneuropsychology when compared to traditional in-person cognitive assessment (Alva et al., 2025; Monteiro et al., 2025), even with older healthy adults and patients with dementia (Hunter et al., 2021). Telehealth, including telepsychogeriatrics and teleneuropsychology, offers a solution to the healthcare accessibility problem. The cost of comprehensive neuropsychological and psychiatric services to all patients would be unbearable (Alzheimer’s disease facts and figures, 2024; Sweet et al., 2002). We thus find ourselves between Scylla—the detrimental effects of mental disorders and cognitive decline on people in low resource areas—and Charybdis—the monetary drainage of the healthcare system.

Community old age mental and cognitive health services in remote low resources areas may prove crucial in the delivery of health care, providing support to people with mental health problems closer to their homes. Remote healthcare, when incorporated into primary care, can achieve increased access to mental and cognitive health services in low-resource settings (Garg et al., 2022), in a broader framework of health digitalization (Majcherek et al., 2024).

A digital transformation and modernisation can help redesign and reorganise core community mental and cognitive health services moving towards a new place-based, integrated multidisciplinary team aligned with primary care networks. The aim of the study is to present detailed data from the INTegRated InterveNtion of pSychogerIatric Care (INTRINSIC) project. The INTRINSIC project was developed in order to address the mental and cognitive imbalance of healthcare needs of older adults residing in low-resources areas of Greece, through integrated digitally enabled mental and cognitive care services, easily accessible by all.

## 2 Methods

### 2.1 Sample cohort

The present analysis was developed, tested and validated using cross-sectional data from the INTRINSIC cohort (Politis et al., 2023; Aggeletaki et al., 2024). The diagnostic and treatment protocols adhered to the principles of the sixth revision of the Declaration of Helsinki and received approval from the Bioethics and Research Ethics Committee of the Eginition Hospital of the University of the National and Kapodistrian University of Athens (approval no. 1,036/31/12/2021, ΑΔΑ 6ΘΞ146Ψ8Ν2-1ΗΙ). Written informed consent was obtained from all beneficiaries or their legally authorized representatives prior to being enrolled in INTRINSIC. All analyses included 1,143 individuals enrolled in the study from 2022 to 2025. Participant characteristics, including demographics and descriptive statistics, are presented in Table 1.

**Table 1:** Participant characteristics for the INTRINSIC cohort.

Characteristics	Descriptive statistics	
Demographics		
Age (*N* = 1,112)	*M (SD)*	72.26 (10.02)
Gender, male (*N* = 1,131)	*n (%)*	359 (32%)
Education (*N* = 1,107)	*n (%)*	Lower Education 424 (39%)
		Secondary Education 314 (28%)
		Higher Education 369 (33%)
Place of residence (*N* = 1,112)	*n (%)*	Andros 179 (16%)
		Chalandritsa 102 (9%)
		Erymanthos 106 (9%)
		Karditsa 24 (2%)
		Katouna 22 (2%)
		Loutraki 70 (6%)
		Soufli 290 (26%)
		Syros 184 (16%)
		Tinos 31 (3%)
		Tripoli 24 (2%)
		Xanthi 80 (7%)
Diagnosis (*N* = 1,111)		
Prior diagnosis of Dementia	*n (%)*	81 (7%)
Prior diagnosis of Depression	*n (%)*	389 (35%)
Prior diagnosis of Anxiety	*n (%)*	424 (38%)
Prior diagnosis of Psychosis	*n (%)*	16 (1%)
Prior diagnosis of Hypertension	*n (%)*	720 (65%)
Prior diagnosis of Dyslipidemia	*n (%)*	602 (54%)
Prior diagnosis of Heart Disease	*n (%)*	337 (30%)
Prior diagnosis of Arthritis	*n (%)*	282 (25%)
Prior diagnosis of Diabetes	*n (%)*	288 (26%)
Prior diagnosis of Thyroid Disorder	*n (%)*	269 (24%)
Prior diagnosis of Parkinson’s disease	*n (%)*	24 (2%)
Medication (*N* = 1,111)		
Antihypertensive	*n (%)*	692 (62%)
Antidepressant	*n (%)*	348 (31%)
Antiarrhythmic	*n (%)*	280 (25%)
Antithrombotic	*n (%)*	284 (25%)
Antidiabetic	*n (%)*	277 (2%)
Thyroid medication	*n (%)*	264 (22%)
Benzodiazepine	*n (%)*	215 (19%)
Antidementia medication	*n (%)*	92 (8%)
Antipsychotic	*n (%)*	73 (7%)
Diuretics	*n (%)*	142 (13%)
Polypharmacy	*n (%)*	439 (40%)
Psychopathology and cognitive scales (*N* = 1,111)		
PHQ-2	*M (SD)*	1.59 (1.71)
GAD-2	*M (SD)*	1.34 (1.65)
Mini-Cog	*M (SD)*	3.67 (1.5)
Mini-Cog Binary, ≤ 2/5	*n (%)*	216 (16%)

*N*, total sample size; n, subsample size (count within a category); M, Mean; SD, Standard Deviation; PHQ-2, Patient Health Questionnaire-2; GAD-2, Generalized Anxiety Disorder-2.

### 2.2 INTRINSIC study

**Figure 1:**
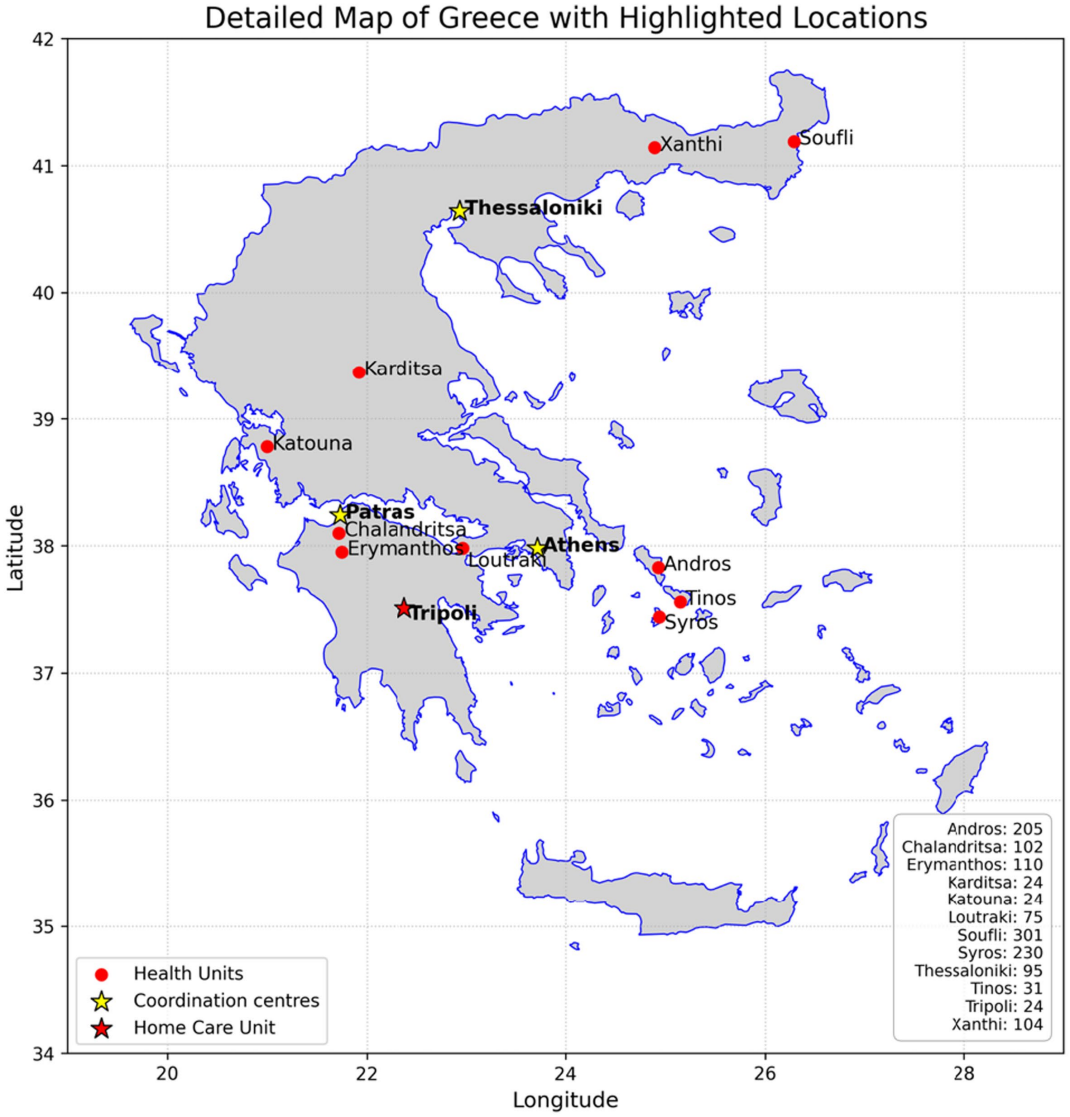
Map of participating healthcare units: primary healthcare units (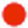), tertiary coordination centers (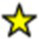), and the home care healthcare unit in Tripoli (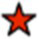).

The recruitment process and study design of INTRINSIC are detailed in previous publications (Politis et al., 2023; Aggeletaki et al., 2024). In brief, an ecosystem of innovative and easy to use information systems has been developed and actively supported, which functions as a single Digital Platform. This technological pillar of the project aims at the electronic interconnection of benefiting populations in rural areas with specialized scientists in psychogeriatric with the important mediation of doctors in remote Health Centers (Figure 1).

The HEllenic Remote MEntal health Services for old-age (HERMES) Digital Platform^1^ integrates innovative Remote Mental Health Services and supports interoperability with other external applications and services. More specifically, it integrates: (a) an innovative System of Telehealth and a Surveillance of Mental and Cognitive Health Risk Factors Survey, (b) a sophisticated e-training system for the members of the project offering self-paced learning courses and webinars in state of the art psychogeriatric topics, a set of sensory health assessments, the Modified Problem Adaptation Therapy (M-PATH) for old-age patients with depression and their carers and (c) an open to the public information and awareness raising materials about mental and cognitive health of old individuals.

As mentioned above, in addition to demographic and medication data, cognitive, behavioral, physical and mental health risk factors are also collected through HERMES.

The Old Age Behavioral Risk Factor Surveillance System (OLA-BRFSS) is a premier system of mental health-related digital survey that collects data from remote Health centers, about Greek old age residents regarding their mental and cognitive health and use of health services. The OLA-BRFSS was established in 2021 as part of the Integrated Psychogeriatric Care Project funded by Hellenic Ministry of Health and designed and implemented in 2022 by the 1^st^ Department of Psychiatry in 8 remote health centers in Greece. By collecting behavioral health risk data at the 11 remote health and community centers, OLA-BRFSS will become a powerful tool for targeting and building integrated mental health promotion activities. The OLA-BRFSS digital survey includes a 20-item questionnaire of clinical significance in older adults: polypharmacy, loneliness, emotional support, hearing loss, falls, weight loss, insomnia, cognitive complaints, traumatic/stressful events, depressive symptoms, anxiety symptoms and cognitive impairment. Psychopathological and cognitive data is collected through the Patient Health Questionnaire-2 (PHQ-2; Kroenke et al., 2003), the Generalized Anxiety Disorder-2 (GAD-2; Sapra et al., 2020) and the Mini-Cog (Fage et al., 2021).

Mini-Cog scores were dichotomized using a threshold of ≥3 (Kroenke et al., 2003; Sapra et al., 2020), for statistical analysis purposes, facilitating the use of advanced classification techniques. Participant characteristics according to Mini-Cog Binary Class stratification are described in Table 2.

**Table 2:** Participant characteristics for the INTRINSIC cohort stratified by Mini-Cog binary class.

Characteristics	Descriptive statistics	Group 1 (Mini-Cog ≥3) *n* = 895	Group 2 (Mini-Cog <3) *n* = 216	Group 1 vs. Group 2 (p)
Demographics				
Age	*M (SD)*	70.69 (9.79)	78.68 (8.22)	**<0.001**
Gender, male	*n (%)*	265 (30%)	85 (38%)	0.662
Education	*n (%)*	Lower Education 302 (34%)	Lower Education 121 (56%)	**<0.001**
		Secondary Education 255 (29%)	Secondary Education 59 (27%)	
		Higher Education 333 (37%)	Higher Education 36 (17%)	
Diagnosis				
Prior diagnosis of Dementia	*n (%)*	30 (3%)	51 (24%)	**<0.001**
Prior diagnosis of Early onset Depression	*n (%)*	108 (12%)	15 (7%)	1.00
Prior diagnosis of Late onset Depression	*n (%)*	189 (21%)	62 (29%)	0.874
Prior diagnosis of Anxiety	*n (%)*	166 (19%)	21 (10%)	0.107
Prior diagnosis of Bipolar Affective Disorder	*n (%)*	3 (0.3%)	1 (0.5%)	1.00
Prior diagnosis of Psychosis	*n (%)*	15 (2%)	1 (0.5%)	1.00
Prior diagnosis of Hypertension	*n (%)*	578 (65%)	142 (66%)	1.00
Prior diagnosis of Dyslipidemia	*n (%)*	493 (55%)	109 (51%)	1.00
Prior diagnosis of Heart Disease	*n (%)*	253 (28%)	84 (39%)	0.124
Prior diagnosis of Arthritis	*n (%)*	224 (25%)	58 (27%)	1.00
Prior diagnosis of Diabetes	*n (%)*	228 (26%)	60 (28%)	1.00
Prior diagnosis of Thyroid Disorder	*n (%)*	220 (25%)	49 (23%)	1.00
Prior diagnosis of Parkinson’s disease	*n (%)*	16 (2%)	8 (4%)	1.00
Prior diagnosis of Stroke	*n (%)*	34 (4%)	14 (7%)	1.00
Prior diagnosis of Cancer	*n (%)*	71 (8%)	18 (8%)	1.00
Medication				
Antihypertensive	*n (%)*	560 (63%)	132 (61%)	1.00
Antidepressant	*n (%)*	267 (30%)	81 (38%)	1.00
Antiarrhythmic	*n (%)*	216 (24%)	64 (30%)	1.00
Antithrombotic	*n (%)*	217 (24%)	67 (31%)	1.00
Antidiabetic	*n (%)*	218 (24%)	59 (27%)	1.00
Diuretic	*n (%)*	95 (11%)	47 (22%)	**<0.001**
Thyroid medication	*n (%)*	201 (23%)	43 (20%)	1.0
Benzodiazepine	*n (%)*	178 (20%)	37 (17%)	1.0
Antidementia medication	*n (%)*	33 (4%)	59 (27%)	**<0.001**
Antipsychotic	*n (%)*	49 (6%)	24 (11%)	0.181
Antiepileptic	*n (%)*	17 (2%)	12 (6%)	0.218
OLA-BRFSS				
Polypharmacy	*n (%)*	322 (36%)	117 (54%)	**<0.001**
Loneliness	*n (%)*	228 (26%)	46 (21%)	1.0
Εmotional support	*n (%)*	684 (76%)	178 (82%)	1.0
Ηearing loss	*n (%)*	255 (26%)	131 (39%)	0.101
Falls	*n (%)*	229 (26%)	77 (36%)	0.160
Weight loss	*n (%)*	102 (11%)	34 (16%)	1.0
Insomnia	*n (%)*	449 (50%)	93 (43%)	1.0
Subjective cognitive complaints	*n (%)*	551 (62%)	166 (77%)	**<0.001**
Traumatic/stressful events	*n (%)*	533 (60%)	101 (47%)	**0.035**
Subjective depressive symptoms	*n (%)*	568 (64%)	139 (64%)	1.0
Subjective anxiety symptoms	*n (%)*	481 (54%)	124 (57%)	1.0
Psychopathology and cognitive scales				
PHQ-2	*M (SD)*	1.57 (1.69)	1.69 (1.78)	1.0
GAD-2	*M (SD)*	1.35 (1.66)	1.34 (1.57)	1.0
Mini-Cog score	*M (SD)*	4.33 (0.81)	1.19 (0.85)	**<0.001**

*n* = subsample size (count within a category), M: Mean, SD: Standard Deviation, p: p-calue, estimated using Welches t-test for continuous variables and chi-square tests for categorical variables. All ps were corrected using the Bonferroni method. Bold ps indicate statistically significant results.

#### 2.2.1 Study variables and outcome

Eighty-eight known risk factors collected through HERMES at initial assessment were analyzed (Supplementary Table S1). These included medical conditions such as hypertension, obesity, infections, heart disease, dyslipidemia, stroke, and Parkinson’s disease. Psychiatric and neuropsychological conditions included a diagnosis of depression, psychosis, anxiety disorder, bipolar disorder, MCI and dementia. Medication history encompassed the use of antidiabetics, antiarrhythmic medications, antihypertensives, thyroid medications, benzodiazepines, lithium, antidepressants, and antipsychotics. PHQ-2 and GAD-2 scores were used in binary form for the Machine Learning (ML) analysis with a cut-off of ≥3. Finally, all items from the previously described 20-item questionnaire documenting clinically significant factors in older adults were included. Further to sample statistics, the classification analysis outcome were the Mini-Cog scores.

#### 2.2.2 Data preprocessing and ML pipeline

Previously, Artificial Intelligence (AI) methods have been applied in healthcare with the aim of supporting decision-making by improving the effectiveness of services through the use of individualized data (Khosravi et al., 2024; Hobensack et al., 2023). The machine learning (ML) model was developed, trained, and evaluated on the INTRINSIC cohort using nested cross-validation (CV), a robust approach that supports both hyperparameter tuning and model selection on a dataset using single-patient cohorts (Cearns et al., 2019). In this setup, the inner CV performs hyperparameter selection, while the outer CV provides an unbiased estimate of model accuracy with CV-based hyperparameter tuning. Because hyperparameter search only accesses a subset of the data defined by the outer CV folds, the risk of overfitting is minimized, yielding a less biased estimate of the tuned ML model’s performance on the dataset (Cawley, 2010).

The pipeline began with data imputation using Multivariate Imputation by Chained Equations (MICE; Azur et al., 2011) implemented via an iterative imputation procedure, which reconstructs missing values by iteratively estimating each incomplete variable from the others until convergence (Zhang, 2016). Imputation models were fitted using Bayesian ridge regression with five imputation iterations and posterior sampling enabled to account for uncertainty. A fixed random seed ensured reproducibility. To prevent information leakage, imputation was performed exclusively within training data for each cross-validation fold. Continuous variables were then standardized to zero mean and unit variance to improve predictive performance (Bishop, 1995). To reduce dimensionality and address multicollinearity, features were selected using Elastic Net regularization, which combines LASSO (L1) and Ridge (L2) penalties to retain important variables while shrinking correlated coefficients (Zou and Hastie, 2005). Models were fitted across a range of L1 mixing parameters (0.7, 0.9, and 1.0) and 200 penalty strengths, using 10-fold cross-validation to select optimal regularisation parameters. To determine the optimal number of predictors, candidate feature set sizes (5, 10, 15, 20, 30, and 50 predictors) were evaluated. For each candidate size, a Random Forest classifier was trained using stratified five-fold cross-validation, and mean area under the receiver operating characteristic curve (ROC AUC) was computed. The feature set size yielding the highest mean ROC AUC was selected for final modelling. A Random Forest (RF) classifier was then trained on the selected features. RF builds an ensemble of decision trees using bagging (bootstrap aggregation), enhancing accuracy, preventing overfitting, enabling automatic feature selection, and generating robust ML models (Zou and Hastie, 2005). It can detect anomalies, identify important features, uncover patterns, and provide insightful graphics. In this study, RF was employed to classify individuals into two Mini-Cog cognitive performance groups. The outer loop consisted of repeated stratified cross-validation to estimate generalisation performance. The inner loop employed stratified 10-fold cross-validation for hyperparameter optimisation using randomised search. Hyperparameters included tree depth, number of trees, node size constraints, and feature subsampling strategies. Class imbalance was addressed using inverse-frequency class weighting. All preprocessing steps were refitted within each training fold. Table 3 summarizes the analytical steps of the Mini-Cog group classification procedure.

**Table 3:** Summary of the Mini-Cog group classification procedure.

Step	Description	Method
1	Handle missing data	MICE
2	Standardize features	Scale to mean of 0 and a standard deviation of 1
3	Feature selection/dimensionality reduction	Elastic Net
4	Classification/Prediction	Random Forest
5	Hyperparameter tuning & validation	Nested CV with 10-fold, 10 repeats (inner loop for tuning, outer loop for testing)
6	Model evaluation	ROC—AUC

**Figure 2:**
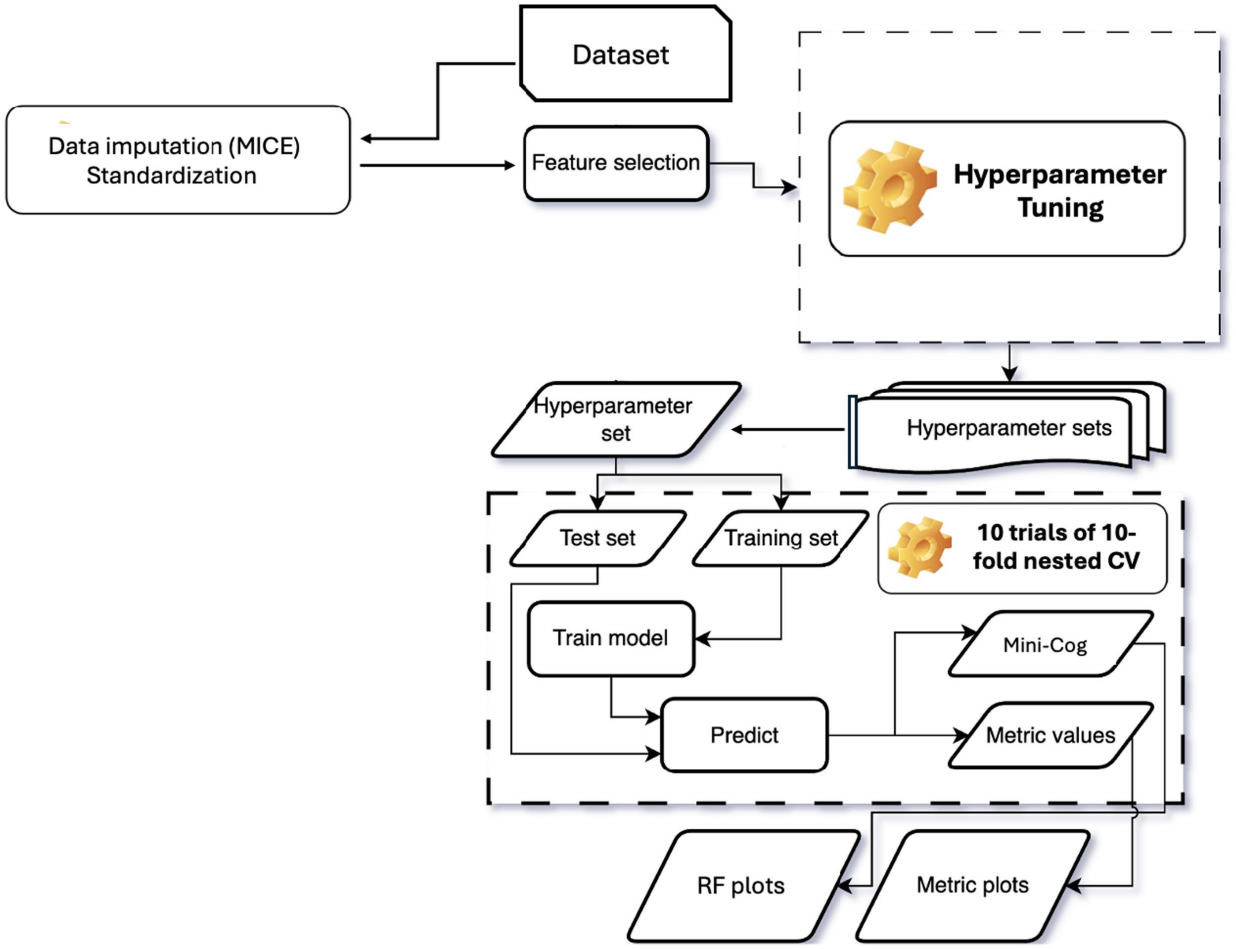
RF model building and Mini-Cog group generation. The dataset was imputed, standardized, and reduced to the selected features using elastic net following hyperparameter tuning. Using the selected hyperparameter set, 10 trials of a 10-fold nested CV were performed to generate Mini-Cog grouping and associated metric values. These values were then used to produce RF plots and metric (ROC–AUC) plots.

The entire process was implemented in a reproducible pipeline (Figure 2). The pipeline was specifically structured to prevent information leakage between training and testing phases. Hyperparameter tuning was performed for RF within in the inner CV loop using 10-fold CV repeated 10 times (Malakouti et al., 2023), optimizing parameters such as regularization strength (Elastic Net; Supplementary Table S2) and number of trees (RF). Model performance was assessed using the Area Under the Receiver Operating Characteristic Curve (ROC-AUC), a measure that quantifies the model’s ability to distinguish between classes across classification thresholds, with values near 1 indicating excellent discriminative power (Cook, 2007). Model performance was further assessed using accuracy, balanced accuracy, F1 score, sensitivity, specificity, predictive values, likelihood ratios, and diagnostic odds ratios. Ninety-five per cent confidence intervals for key metrics (ROC AUC, accuracy, balanced accuracy, and F1 score) were estimated using non-parametric bootstrap resampling (2,000 iterations) of pooled out-of-fold predictions. All random seeds, hyperparameters, and feature selection decisions were recorded to ensure full reproducibility.

A similar rationale for the aforementioned approaches to developing and evaluating ML models for dementia prediction can be found in Nori et al. (2019) and Twait et al. (2023), and references therein. For a recent review of algorithms using electronic health record (EHR) data to identify patients with Alzheimer’s disease and related dementias (ADRD) and to advance their use in research and clinical care, see Walling et al. (2023).

All analyses were performed using Python 3.11 using scikit-learn, scipy, statsmodels, pandas, and matplotlib libraries. The full code used in the analyses is available from the corresponding author upon reasonable request.

## 3 Results

### 3.1 Sample descriptive characteristics

The total number of individuals included in the analyses was 1,143, of whom 359 (32%) were male and average age was 72.26 years (SD = 10.02). Regarding physical illnesses, 720 (65%) participants had a diagnosis of hypertension, 602 (54%) had dyslipidemia and 337 (30%) had heart disease. With respect to psychiatric and cognitive disorders, 81 (7%) had a prior diagnosis of dementia, 424 (38%) had a diagnosis of anxiety, and 389 (35%) had a diagnosis of depression. Mean PHQ-2 was 1.59, with 288 individuals (26%) scoring above the cut-off of 3. The mean GAD-2 score was 1.34, while 226 individuals (20%) scoring above the cut-off of 3. Finally, 216 (16%) participants had a Mini-Cog below the cut-off score, indicating possible cognitive decline. See Table 1 for details.

Statistical comparisons between the two Mini-Cog groups (see Table 2), revealed significant differences. Individuals in Group 2 (Mini-Cog <3) were older, had a lower level of education, were more likely to have a diagnosis of dementia and take related medication, were more likely to take diuretics, were more likely to be on more than five medications (polypharmacy; Pazan and Wehling, 2021), and subjective cognitive complaints were more frequently reported. Finally, traumatic and stressful events were more often reported by individuals whose Mini-Cog performance was above the cut-off score.

### 3.2 Binary Mini-Cog classification analysis

To perform an in-depth, multivariate classification analysis, a Random Forest (RF) classifier was applied (Breiman, 1996). RF is a supervised learning classification algorithm that consists of multiple classifiers, called decision trees, built independently. The decision about which class each participant belongs to is based on the majority of the results of these classifiers (i.e., majority voting). RF is particularly suitable for large datasets due to its ability to handle numerous input variables, manage missing data, and identify variable associations. Additionally, it provides data insights through variable importance rankings (Shaik and Srinivasan, 2019) and has been successfully applied in psychiatry, psychology and the medicine (Sarica et al., 2017; Bracher-Smith et al., 2020; Dimitriadis and Liparas, 2018; Del Casale et al., 2023; Kaur and Sharma, 2019; Lima and De Castro, 2019; Yarkoni and Westfall, 2017; Wallace et al., 2023; Speiser et al., 2019; Prinzi et al., 2024). The ML pipeline used is illustrated in Figure 2.

Fifteen variables out of eighty-three were selected by the Elastic Net (Supplementary Table S3), following a process of hyperparameter optimization, with positive or negative weights assigned to each. Positive weights favored Group 2 (Mini-Cog <3), whereas negative weights favored Group 1 (Mini-Cog ≥3). This set of variables was then used by the RF algorithm and they were ranked according to their relative importance from most important to least important (Elastic Net weighting), as follows: (1) use of antidementia medication (+), (2) prior diagnosis of dementia (+), (3) subjective complaint of recent memory (+), (4) subjective complaint of mental clouding (+), (5) being retired from work (+), (6) prior diagnosis of an anxiety disorder (−), (7) documented polypharmacy (+), (8) hearing problems (+), (9) the use of diuretic medication (+), (10) experiencing stressful events (−), (11) complaints of insomnia symptoms (−), (12) ability to provide personal means of transport (−), (13) a history of falls (+), (14) ability to exercise regularly (−), and (15) living in the same household with their children (+).

**Figure 3:**
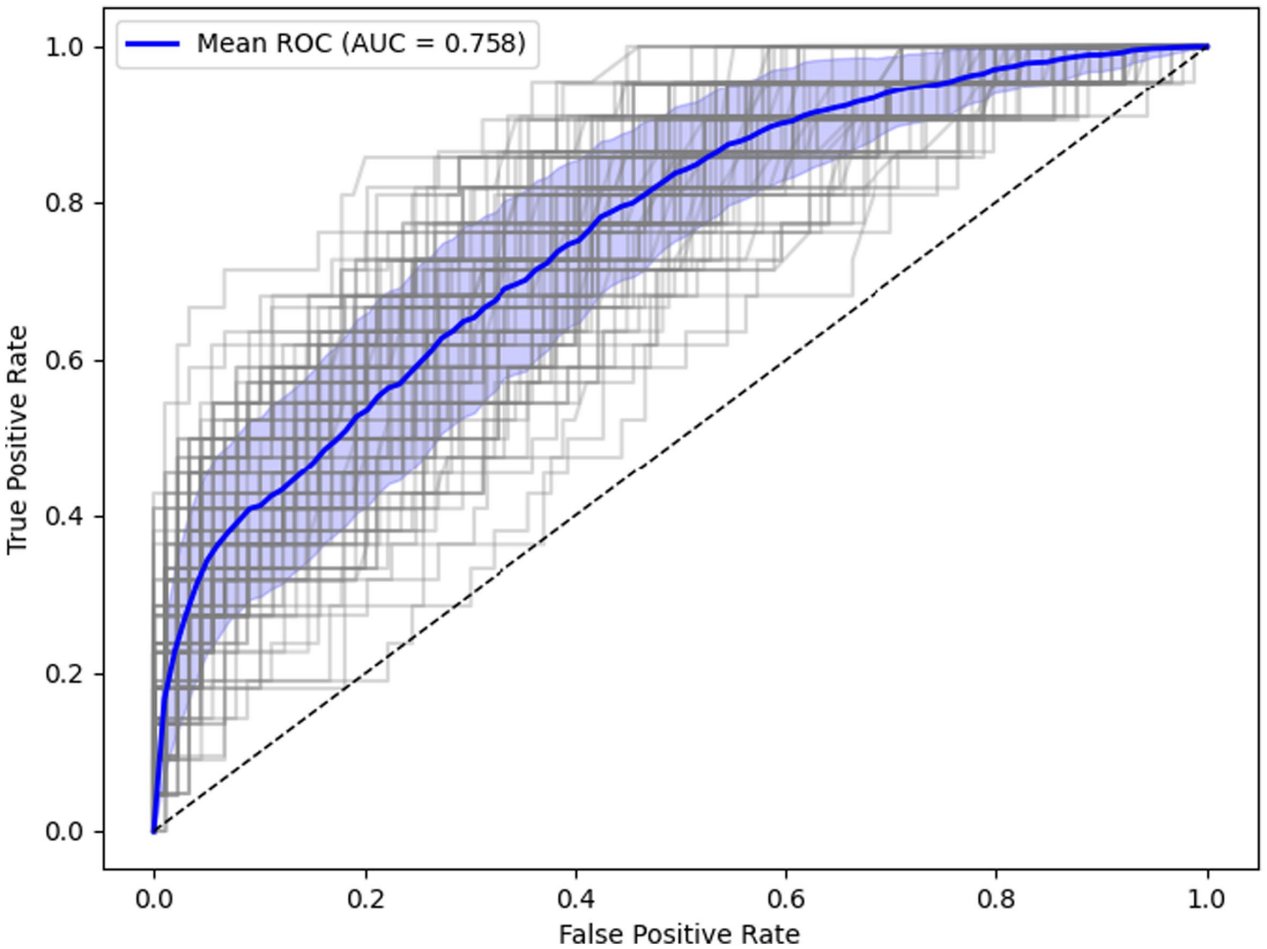
ROC curve.

The model achieved a ROC-AUC of 0.76 (Figure 3 and Table 4), with a balanced accuracy (BAC) of 0.65. A negative predictive value (NPV) of 0.87 indicates strong performance in identifying Group 1 cases, whereas a positive predictive value (PPV) of 0.40 reflects limited precision in confirming Group 2 classification. All performance metrics are included in Table 4. Learning curve diagram, variability of selected features and confusion matrix can be found in Supplementary Figures S1–S3.

**Table 4:** Outer loop cross validation metrics.

Machine learning model	Model Selection (Outer Loop Testing)—10 repeats of 10-fold CV										
	AUC	BAC	Acc	Sens	Spec	PPV	NPV	PLR	NLR	DOR	F1-score
RF model	0.76 (±0.06)	0.65 (±0.03)	0.75 (±0.03)	0.50 (±0.12)	0.81 (±0.04)	0.40 (±0.07)	0.87 (±0.02)	2.82 (±0.84)	0.61 (±0.14)	5.15 (±2.54)	0.45 (±0.07)

Metric abbreviations: AUC, area-under-the-curve; BAC, balanced accuracy; Acc, accuracy; Sens, sensitivity; Spec, specificity; PPV, positive predictive value; NPV, negative predictive value; PLR, positive likelihood ratio; NLR, negative likelihood ratio; DOR, diagnostic odds ratio. The values in parentheses indicate the standard deviation of the metric across the repeated CV folds.

## 4 Discussion

The INTRINSIC study has thus far enabled access to a substantial community population that underwent neurocognitive screening with the aim to identify individuals exhibiting cognitive decline. A multitude of factors were systematically collected at this stage, to investigate possible associations with neurodegenerative disorders. Cross-sectional analysis of correlations between these factors and cognitive functioning can aid towards organizing timely therapeutic interventions, also revealing interactions with demographic and clinical features. Central to this effort is the integrated electronic system utilized, providing a digital hub for communication, information, data management and patient monitoring. Through this framework clinicians are able to identify low prevalence conditions such as Parkinson’s disease, and explore the fine grain of clinical conditions, including late and early depressive disorder.

The current project can provide clinically useful information based on descriptive statistics alone. For example, there is a clear sex bias, i.e., there were much more women visiting the sites of INTRINSIC, compared to men. Even though this has been reported in the literature and could be attributed to several factors (Bertakis et al., 2000; Barsky et al., 2001), the identification of such a sex-dependent imbalance in healthcare utilization, can serve as a criterion for planning group-focused awareness-raising activities for the community. Similarly, differences between sites with regard to mean age of beneficiaries or proportions of actually cognitively impaired individuals visiting the clinical setting (in contrast to those who had subjective complaints without any objective signs of impairment), could be also helpful in that direction. Moreover, we have found a significant proportion of individuals who had an indication of cognitive decline, despite lack of prior dementia diagnosis. In a recent meta-analysis, Lang and colleagues have shown that the prevalence of undiagnosed dementia is quite high, related to several factors including socioeconomic status, sex, educational level, and age (Lang et al., 2017). In our sample, a considerable portion of participants had lower education. Thus, the indications of undetected dementia could be attributed to less years of formal schooling, among other factors. This has been already documented in the literature (Savva and Arthur, 2015; Amjad et al., 2018) and poses a significant impediment to diagnostic equity. That is where projects like INTRINSIC could aid in amending such inequalities, by raising awareness via educational activities and detecting patients with dementia in a primary care framework.

The ML algorithm employed in this study revealed some interesting factors that are associated with the Mini-Cog scores. Eight features were found to be positively associated with low Mini-Cog scores, namely, a subjective complaint of recent memory impairment, a subjective complaint of mental clouding, being retired from work, polypharmacy, hearing impairment, diuretic medication use, history of falls, and residing with adult children to live in the same household. All of these have previously been recognized to participate in the trajectory of dementia (Mendonça et al., 2016; Basagni and Navarrete, 2025; Hallam et al., 2021; Dufouil et al., 2014; Leelakanok and RR, 2019; Powell and Reed, 2024; Lad et al., 2024; Liu et al., 2022; Tully et al., 2016; Fernando et al., 2017; Desai et al., 2020). The convergence of machine learning-derived results with existing evidence base strengthens the validity of these associations and underscores the multifactorial nature of cognitive decline.

Five variables demonstrated a positive association with higher Mini-Cog scores. A prior diagnosis of an anxiety disorder, experiencing stressful events, self-reported symptoms of insomnia, access to personal means of transport and being able to exercise physically regularly. The protective effects of maintained mobility and regular physical activity against cognitive decline have been well-established in previous research (Alty et al., 2020; Ahlskog et al., 2011). Interestingly, previous studies have found that clinical anxiety, stress and insomnia are positively correlated to dementia (Santabárbara et al., 2019; Gulpers et al., 2016; de Almondes et al., 2016; Hung et al., 2018). It could be argued, however, that unlike previous investigations that primarily examined clinical cohorts, our sample stemmed from community individuals, representing a different demographic profile, potentially affecting these variables. The results of this study align with previous research demonstrating negative associations between anxiety, stressful life events, and Alzheimer’s dementia incidence, though findings in this area remain mixed (Franks et al., 2021; Santabárbara et al., 2019). These findings suggest that the relationship between these features and the progression of cognitive function may be more context-dependent than previously understood, requiring further investigation of the underlying mechanisms. Integrating these findings in clinical settings would require further exploring the application of the ML algorithm in a variety of populations to test replicability and generalizability of these results. This process includes several caveats, such as access to care, data noise, missing values and indication bias (Khosravi et al., 2024).

In the context of primary care, efficient and reliable predictive models for cognitive decline are particularly valuable because clinicians often face constraints in time, resources, and patient burden. The INTRINSIC project, which assesses a wide range of cognitive health–related risk factors—including social and emotional support, lifestyle behaviors, sensory impairments, mental health history, and direct cognitive screening, among others—provides a rich multidimensional dataset that can inform early dementia detection. Within this framework, efficient study design is crucial given the high cost of data collection. As a future direction, we aim to leverage this rich, multidimensional dataset to develop a database-driven design selection scheme that integrates metaheuristics and data mining to identify the most influential variables for MiniCog prediction and early dementia detection. This approach would enable optimal design retrieval from such a large observational dataset, enhancing model precision, interpretability, and clinical relevance. Building on the Design of Experiments (DOE) paradigm (Pumplün et al., 2005), we plan to identify the most informative observations and features, initially using rule mining techniques to uncover hidden relationships, followed by the application of a genetic algorithm to construct an optimal supersaturated design that isolates the vital few variables with run-size economy (Parpoula et al., 2014). This alternative approach to variable selection (Parpoula et al., 2014), applied to a multidimensional database of observations, enables the utilisation of only a small fraction of the available runs, making statistical analysis of large databases computationally feasible and cost-effective. From a primary care perspective, the ultimate goal is to identify a parsimonious set of predictors that maintain predictive accuracy while minimizing the number of assessments required. In this regard, the planned future approach of database-driven design selection aligns closely with clinical priorities. Moreover, this strategy could support both the identification of important individual features and the exploration of meaningful feature combinations, paving the way for robust, interpretable, and clinically actionable models for cognitive health screening. Such models could inform clinical support systems focused on predictive classification and early intervention.

There are a few limitations of this study. First, due to the nature of the data collection pipeline, there are key data missing for some participants (as shown in Table 1), which are impossible to retrieve because of anonymization. Related to that, we should also mention possible measurement errors due to multiple assessors in various sites. In addition, all individuals included were of Greek descent, this leads to exclusion of minority populations. Sites are distributed throughout the mainland and the Greek islands, with recruitment numbers varying depending on various factors, such as the size of the local population and enrollment strategy. Second, there is a lack of comprehensive neuropsychological assessment and full, structured psychiatric evaluation. However, we argue that, at least to some degree, this is considered an inevitable drawback, when attempting to establish a low-resource, multi-site, primary care network. In this sense, a low-cost, broad and quick evaluation can help us identify the specific individuals in need of a thorough psychiatric and/or neuropsychological assessment, thus protecting resources, which are already low in such sites. Third, there is a sample bias, regarding the manner in which beneficiaries visited the sites. Nevertheless, with advancing time, and with more data accumulated, subsamples will be more representative of the populations the sites correspond to. Regarding the ML methodology, (Bleeker et al., 2003; Ramspek et al., 2021) as found in prior studies, the use of a multivariate model in a cross-sectional context could allow for the identification of a potential signature of the factors that correlate to cognitive functioning in this patient cohort (Zhou et al., 2023; Li et al., 2023). As indicated by the relatively low PPV value, further exploration of the generalizability of this model could allow for adaptation and potential application in the clinical context. An external validation sample would provide robust evidence of replicability of results, testing the algorithm’s reproducibility. In this case, the nature of the data collected used impedes efforts to identify a compatible external sample. Albeit the large number of the sample allows for a nested CV method to provide an indication of the model’s external performance (Bleeker et al., 2003). Once a predictive algorithm is found to successfully replicate in an external sample, a cohort with different patient characteristics can be utilized to test transportability (Ramspek et al., 2021).

In conclusion, this paper presents for the first time a large-scale study on INTRINSIC, including multiple sites and integrating psychiatric, cognitive, medical, as well as sociodemographic variables in state-of-the-art ML models. Our findings provide insights into the complex interrelationships of factors affecting cognitive status in old individuals. We further argue that INTRINSIC can serve as a benchmark for the incorporation of psychiatric and neuropsychological care of old people, especially those suffering from dementia or MCI, in the broader framework of primary healthcare, in an effort to remediate the issues of access and diagnostic inequality discussed in this paper.

## Data Availability

The datasets presented in this article are not readily available because the original contributions presented in the study are included in the article/Supplementary material, further inquiries can be directed to the corresponding authors. Requests to access the datasets should be directed to apolitis@med.uoa.gr.

## Footnotes

1
https://hermes-tk.med.uoa.gr/
