# Supplementary material for: INTegRated InterveNtion of pSychogerIatric Care: real-world application and implementation of an advanced integrated telehealth system incorporating machine learning
Source: Front Psychol. 2026 Jan 14;16:1696407. doi: 10.3389/fpsyg.2025.1696407 (PMC12847031; doi:10.3389/fpsyg.2025.1696407)

**Supplementary Appendix**

**INTegRated InterveNtion of pSychogerIatric Care: Real-World Application and Implementation of an Advanced Integrated Telehealth System Incorporating Machine Learning**

Rigas F. Soldatos^1^, Dimitrios Kasselimis^2,3^, Christina Parpoula^2^, Eleni Konidari^4^, Vassilis Dimitriou^1^, Everina Katirtzoglou^1^, Dimitris Kiosses^5^, Konstantinos Tsibanis^6^, Anastasia Konsta^7^, Theofanis Vorvolakos^8^, Panagiotis Alexopoulos^9^, Antonios Politis^1^

**Methods.**

**Table 1.** Table of all clinical predictors from INTRINSIC entered into the binary Mini-Cog model for feature selection

**Table 2.** Table of Elastic Net Hyperparameters

**Table 3.** Predictors sorted by weight by the Elastic Net during training and Spearman

correlation coefficients.

**Table 4. Hyperparameters.** Full Pipeline Hyperparameters.

**Figure 1.** Learning Curve diagram of the Mini-Cog classifier

**Figure 2.** Variability of the selected features in the Elastic Net for the classification model.

**Figure 3.** Confusion matrix indicating classification accuracy in the classification model.

**Supplementary Methods**

**Supplementary Table S1.** Table of all clinical predictors from INTRINSIC entered into the binary Mini-Cog model for feature selection

| **Variable Name** | **Description** |
| --- | --- |
| Hypertension | History of |
| Cancer | History of |
| Obesity | History of |
| Diabetes | History of |
| Chronic_Respiratory_Failure | History of |
| Urinary_Tract_Infection | History of |
| Arthritis_Joint_Pain | History of |
| Autoimmune_Disease | History of |
| 'Heart_Disease' | History of |
| 'Kidney_Disease' | History of |
| 'Thyroid_Disorder' | History of |
| 'Dyslipidemia' | History of |
| 'Parkinsons_Disease' | History of |
| 'Stroke' | History of |
| 'COVID' | History of |
| 'Dementia' | Diagnosis of |
| 'Early_Onset_Depression' | Diagnosis of |
| 'Late_Onset_Depression' | Diagnosis of |
| 'Mild_Cognitive_Impairment' | Diagnosis of |
| 'Psychosis' | Diagnosis of |
| 'Anxiety_Disorders' | Diagnosis of |
| 'Bipolar_Disorder' | Diagnosis of |
| 'Residence_City' | Demographics |
| 'Residence_Village' | Demographics |
| 'Marital_Status_Divorced' | Demographics |
| 'Marital_Status_Married' | Demographics |
| 'Marital_Status_Single' | Demographics |
| 'Marital_Status_Widowed' | Demographics |
| 'Children_Household_Children_live_in_the_same_city' | Demographics |
| 'Children_Household_Children_live_together' | Demographics |
| 'Living_Arrangement_Lives_Alone | Demographics |
| 'Living_Arrangement_Lives_with_Others' | Demographics |
| 'Employment_Status_Farmer' | Demographics |
| 'Employment_Status_Housewife' | Demographics |
| 'Employment_Status_Livestock_Farmer' | Demographics |
| 'Employment_Status_Private_Employee' | Demographics |
| 'Employment_Status_Public_Employee' | Demographics |
| 'Employment_Status_Retired' | Demographics |
| 'Employment_Status_Self_Employed', | Demographics |
| 'Employment_Status_Teacher' | Demographics |
| 'Employment_Status_Unemployed' | Demographics |
| 'Employment_Status_Worker' | Demographics |
| 'Diuretics' | Medication |
| 'Diabetes_Medications' | Medication |
| 'Epilepsy_Medications' | Medication |
| 'Antiarrhythmic_Medications' | Medication |
| 'Hypertension_Medications' | Medication |
| 'Antithrombotic_Medications' | Medication |
| 'Thyroid_Medications' | Medication |
| 'Parkinson’s_Medications' | Medication |
| 'Vitamins_Supplements' | Medication |
| 'Haloperidol' | Medication |
| 'Akineton' | Medication |
| 'Atypical_Antipsychotics' | Medication |
| 'Benzodiazepines' | Medication |
| 'Lithium' | Medication |
| 'Antidementia_Drugs' | Medication |
| 'Antidepressants' | Medication |
| 'Social_Emotional_Support' | OLA-BRFSS |
| 'Life_Satisfaction' | OLA-BRFSS |
| 'Stressful_Events' | OLA-BRFSS |
| 'Discomfort' | OLA-BRFSS |
| 'Polypharmacy' | OLA-BRFSS |
| 'Falls' | OLA-BRFSS |
| 'Stigma' | OLA-BRFSS |
| 'Hearing_Problems' | OLA-BRFSS |
| 'Physical_Exercise' | OLA-BRFSS |
| 'Weight_Loss' | OLA-BRFSS |
| 'Alcohol_Use' | OLA-BRFSS |
| 'Cigarette_Use' | OLA-BRFSS |
| 'Vulnerability' | OLA-BRFSS |
| 'Insomnia' | OLA-BRFSS |
| 'Cognitive_Decline' | OLA-BRFSS |
| 'Forget_More_Than_Last_Year' | OLA-BRFSS |
| 'Forget_Current_Things' | OLA-BRFSS |
| 'Not_Remembering_Names' | OLA-BRFSS |
| 'Difficulty_Finding_Words' | OLA-BRFSS |
| 'Mind_Foggy' | OLA-BRFSS |
| 'Forget_Where_Put_Things' | OLA-BRFSS |
| 'Accessibility' | OLA-BRFSS |
| 'History_of_Depression' | OLA-BRFSS |
| 'Depression_Less_Interest_Pleasure' | OLA-BRFSS |
| 'Depression_Sorrow_Sadness_Despair' | OLA-BRFSS |
| 'PHQ_Score_Cut_Off' | PHQ |
| 'History_of_Anxiety_Disorder' | OLA-BRFSS |
| 'Anxiety_Nervousness_Impatience' | OLA-BRFSS |
| 'Anxiety_Difficult_To_Manage_Worry' | OLA-BRFSS |
| 'GAD_Score_Cut_Off'] | GAD |

**Supplementary Table S2. Hyperparameters.** Elastic Net Hyperparameters.

| **selected_l1_ratio_** | **selected_alpha_** | **enet_cv** | **enet_l1_ratio_candidates** | **enet_n_alphas** |
| --- | --- | --- | --- | --- |
| 0.7 | 0.015219555025448845 | 10 | [0.7, 0.9, 1.0] | 200 |

**Supplementary Table S3.** Predictors sorted by weight by the Elastic Net during training and Spearman

correlation coefficients. Spearman correlation coefficients can be used in interpreting feature rank ordering.

| **Feature** | **Spearman’s r** | ***p* value bonferroni*** | **effect_size** | **Elastic Net Weight** |
| --- | --- | --- | --- | --- |
| Antidementia Drugs | 0.33929555757218444 | 3.706139681928465e-30 | Moderate | 0.08442591368611174 |
| Dementia | 0.30838881335271245 | 9.926086097332527e-25 | Moderate | 0.034805970430454074 |
| Forget Current Things | 0.19093034535054748 | 2.0988817504096515e-09 | Weak | 0.03450325096123035 |
| Mind Foggy | 0.17287057242869777 | 9.989328110452079e-08 | Weak | 0.03245316901269295 |
| Employment Status Retired | 0.15427966236542787 | 3.5572048346503303e-06 | Weak | 0.017782977403372376 |
| Polypharmacy | 0.14724163482715452 | 1.2386727488844327e-05 | Weak | 0.015310582539387906 |
| Diuretics | 0.13209976483291908 | 0.0001497590197709928 | Weak | 0.023634027669311723 |
| History of Anxiety Disorder | -0.10503113352190051 | 0.006807430519553891 | Weak | -0.025950229116588442 |
| Accessibility | -0.10370029197492983 | 0.008044154881268166 | Weak | -0.017860360561513838 |
| Stressful Events | -0.10229033143839818 | 0.009580329645733843 | Weak | -0.012092514708983475 |
| Hearing Problems | 0.09326331285491944 | 0.027882464102816595 | Negligible | 0.01062112224316982 |
| Falls | 0.08913430595480926 | 0.044153455583566925 | Negligible | 0.008758636879460754 |
| Physical Exercise | -0.0827285505976892 | 0.08693932018235846 | Negligible | -0.009581591121726386 |
| Insomnia | -0.05630888688226174 | 0.9093246813554555 | Negligible | -0.011466487163916399 |
| Children Household Children live together | 0.0489825539970124 | 1.0 | Negligible | 0.01814061848110092 |

* *p* values corrected using Bonferroni correction

**Table S4. Hyperparameters.** Full Pipeline Hyperparameters.

**Supplementary Figure S1.** Learning Curve diagram of the Mini-Cog classifier


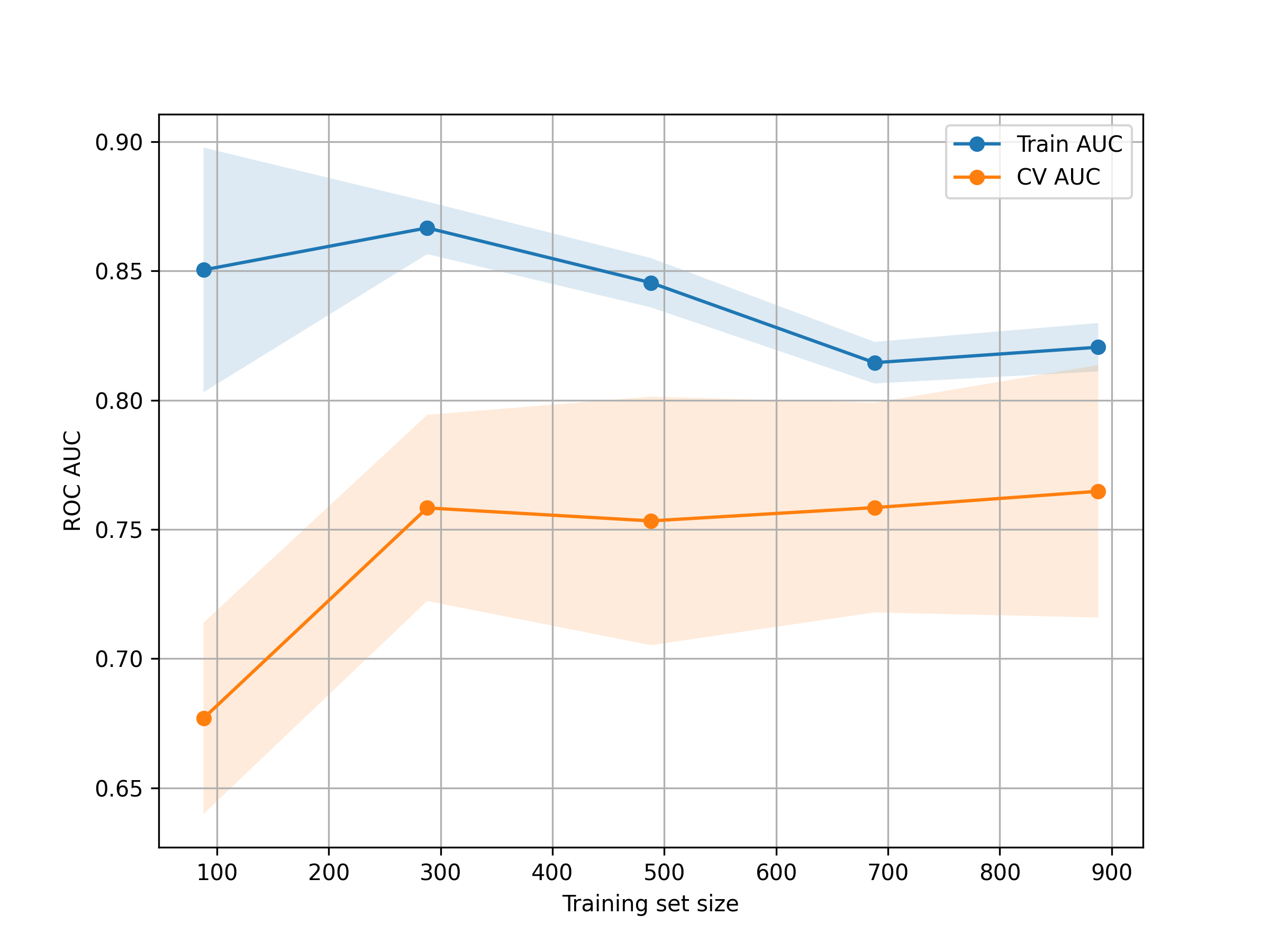


**Supplementary Figure S3.** Variability of the selected features in the Elastic Net for the classification model.


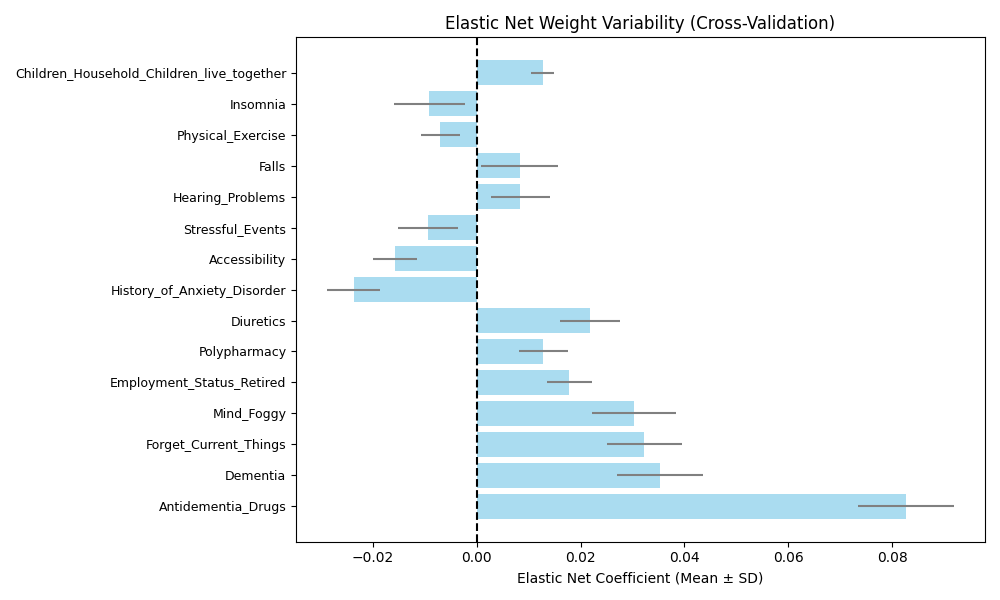


**Supplementary Figure S4.** Confusion matrix indicating classification accuracy in the classification model.


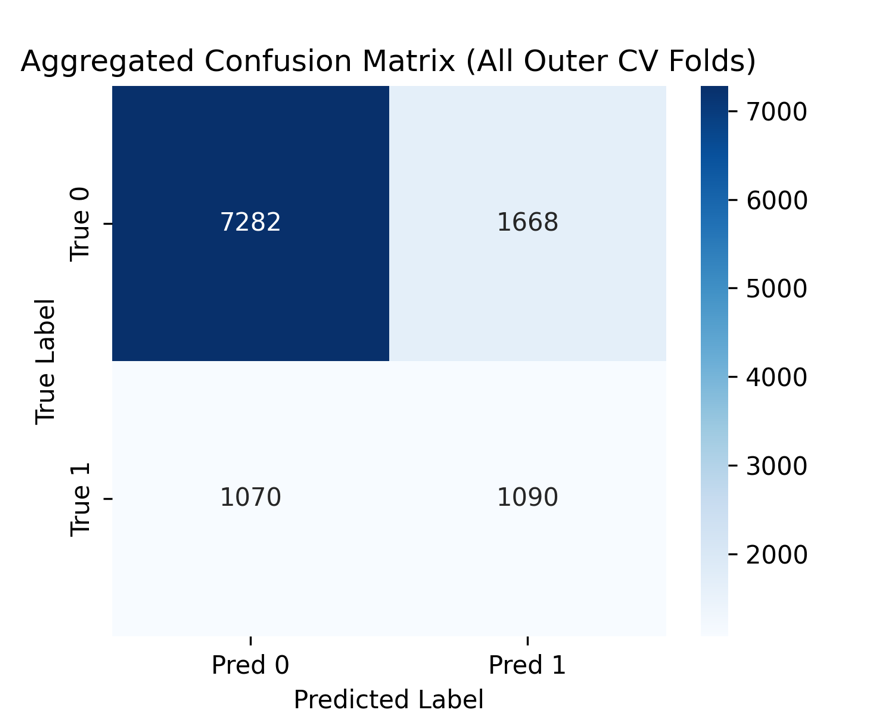

Supplement: Supplementary file 1 [file Table_1.docx]
